# Prevalence of pathogens in ticks collected from humans through citizen science in Belgium

**DOI:** 10.1186/s13071-019-3806-z

**Published:** 2019-11-21

**Authors:** Tinne Lernout, Nick De Regge, Katrien Tersago, Manoj Fonville, Vanessa Suin, Hein Sprong

**Affiliations:** 1 Sciensano, Belgian Institute for Health, Brussels, Belgium; 2 Centre for Infectious Disease Control, National Institute for Public Health and Environment (RIVM), Bilthoven, The Netherlands

**Keywords:** Ticks, Tick-borne pathogens, Humans, Citizen science, Lyme borreliosis

## Abstract

**Background:**

In order to evaluate the risk of human exposure to tick-borne pathogens in Belgium, a study on the prevalence of several pathogens was conducted on feeding ticks removed from humans in 2017.

**Methods:**

Using a citizen science approach based on an existing notification tool for tick bites, a sample of ticks was collected across the country. Collected ticks were screened by PCR for the presence of the following pathogens: *Anaplasma phagocytophilum*, *Babesia* spp., *Borrelia burgdorferi* (*sensu lato*), *Borrelia miyamotoi*, *Neoehrlichia mikurensis*, *Rickettsia helvetica* and tick-borne encephalitis virus (TBEV).

**Results:**

In total, 1599 ticks were included in the sample. The great majority of ticks belonged to *Ixodes ricinus* (99%); other tick species were identified as *Ixodes hexagonus* (0.7%) and *Dermacentor reticulatus* (0.3%). *Borrelia burgdorferi* (*s.l.*) was detected in 14% of nymphs and adult ticks. Adult ticks (20%) were more likely to be infected than nymphs (12%). The most common genospecies were *B. afzelii* (52%) and *B. garinii* (21%). Except for TBEV, the other tick-borne pathogens studied were all detected in the tick sample, although at a lower prevalence: 1.5% for *Babesia* spp.; 1.8% for *A. phagocytophilum*; 2.4% for *B. miyamotoi*; 2.8% for *N. mikurensis*; and 6.8% for *R. helvetica*. *Rickettsia raoultii*, the causative agent of tick-borne lymphadenopathy, was identified for the first time in Belgium, in two out of five *D. reticulatus* ticks. Co-infections were found in 3.9% of the examined ticks. The most common co-infection was *B. burgdorferi* (*s.l*.) + *N. mikurensis.*

**Conclusions:**

Although for most of the tick-borne diseases in Belgium, other than Lyme borreliosis, no or few cases of human infection are reported, the pathogens causing these diseases were all (except for TBEV) detected in the tick study sample. Their confirmed presence can help raise awareness among citizens and health professionals in Belgium on possible diseases other than Lyme borreliosis in patients presenting fever or other non-characteristic symptoms after a tick bite.
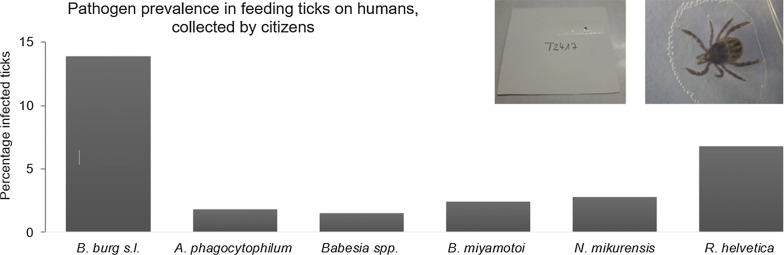

## Background

Ticks are important vectors of infectious diseases affecting human health. The most common tick-borne disease in Europe is Lyme borreliosis, caused by bacteria of the *Borrelia burgdorferi* (*sensu lato*) complex. The bacteria may infect different organs, resulting in skin, neurological, musculoskeletal or cardiac manifestations in humans. In Europe, at least five different genotypes are pathogenic to humans: *B. afzelii*, *B. garinii*, *B. burgdorferi* (*sensu stricto*), *B. bavariensis* and *B. spielmanii* [1–3]. The contribution to disease of some other genospecies, such as *B. valaisiana* and *B. lusitaniae*, is not clear [1, 4]. In Belgium, the incidence of Lyme borreliosis is estimated at 103 per 100,000 (95% UI 87–120), based on a meta-analysis [5].

Although reported far less frequently, infections with other pathogens transmitted by *Ixodes ricinus*, such as tick-borne encephalitis virus (TBEV), *Anaplasma phagocytophilum*, *Borrelia miyamotoi*, *Neoehrlichia mikurensis*, *Rickettsia* spp. and several *Babesia* spp., may cause human disease as well [6, 7]. Approximately two-thirds of human infections with TBEV are asymptomatic. In clinical cases, tick-borne encephalitis (TBE) often has a biphasic course, with a first phase presenting as a flu-like illness, followed in one-third of the patients by a second phase with central nervous system involvement (such as encephalitis or meningitis) [8]. Serological studies in animals suggest that TBEV has been circulating at a low level for at least several years in Belgium and infections in humans were expected to occur [9]. In 2018, the two first TBE cases with possible/probable autochthonous infection were reported [10]. Human infections with *Anaplasma phagocytophilum* are often asymptomatic or present as a mild self-limiting flu-like illness, but severe complications (e.g. opportunistic infections) and fatal infections are possible. In Belgium, confirmed cases of human granulocytotropic anaplasmosis are rare, but considerable underdiagnosis is suspected due to difficulties in diagnosis and lack of awareness among physicians [11].

The relapsing fever spirochete *B. miyamotoi* has recently been identified as pathogenic for humans, causing a nonspecific flu-like illness, with possible severe disease in immunocompromised patients [12]. Only a few cases have been reported in Europe, but the species has been detected at low prevalence in ticks throughout Europe [13]. No human infection has been detected in Belgium so far. *Ixodes ricinus* can also transmit *Neoehrlichia mikurensis*, also known as “*Candidatus* Neoehrlichia mikurensis”. This species is widespread and has been found in questing ticks in at least 20 European countries [14]. It was first described as a human pathogen in a Swedish patient in 2010 and since then only a limited number of human infections have been described, often in immunocompromised patients [14]. There have been no reports of human disease in Belgium.

*Babesia* spp. mainly cause disease in livestock, domestic and wild animals. Infection in humans is often asymptomatic or mild but severe disease has been reported, mainly in asplenic or immunocompromised individuals [15]. Three species have been reported to cause disease in humans in Europe, *B. divergens*, *B. venatorum* (sp. EU1) and, to a lesser extent, *B. microti* [15]. Although no clinical cases of babesiosis have been reported in Belgium so far, specific antibodies for the three European species have been detected in blood from patients with a history of a tick bite and clinical symptoms (mainly fever) [16]. Several *Rickettsia* species are transmitted by ticks in Europe, including *R. conorii* transmitted by *Rhipicephalus sanguineus* (*s.l.*) ticks, *R. helvetica* and *R. monacensis* by *Ixodes ricinus* and *R. slovaca* and *R. raoultii* by *Dermacentor marginatus* and *Dermacentor reticulatus* [17]. The most apparent rickettsial disease in Europe is the Mediterranean spotted fever caused by *R. conorii*, found mostly in southern and eastern Europe [17]. *Rickettsia slovaca* and *R. raoultii* have more recently been associated with human disease, a syndrome characterized by scalp eschars and cervical lymphadenopathy [17]. The pathogenicity of *R. helvetica* is questionable, but infected patients with an atypical and mild clinical picture (fever, skin rash and muscle aches) and some more serious illnesses have been described in Europe [17]. In Belgium, about 20 cases of rickettsioses (tick-borne and others) are reported every year; the rickettsia species isolated from patients are *R. conorii* and *R. africae*, related to travelling in the Mediterranean region and South Africa [18].

Since most of the above diseases present with mild and non-characteristic symptoms, it is difficult to assess their public health risk and burden. As a first step, it is important to have information on the geographical distribution and prevalence of these pathogens in ticks to evaluate the risk of exposure through tick bites and consequently the risk of disease.

In Belgium, most of the previously conducted research concerned questing ticks collected from relatively small areas during a short time period, or ticks collected from animals [19–26]. However, the evaluation of pathogens in feeding ticks represents the risk of human exposure better than studies in questing ticks. Therefore, the present study on the prevalence of several tick-borne pathogens in ticks was conducted on ticks removed from humans in Belgium, in a larger sample collected over several months and all over the country, with the participation of citizens. The choice of pathogens included was based on previous evidence of their presence in *I. ricinus* ticks and their association with human diseases in Europe.

## Methods

### Sample collection and identification

In March 2017, citizens were invited through a press release to send in ticks that were attached to their body (or to another person) to the Belgian Health Institute Sciensano, between April 1st and October 31st [27]. They were asked to attach the tick on a piece of paper or postal card, with transparent tape. In order to allow collection of additional data on the tick bite occurrence, a questionnaire had to be filled in through the website TekenNet. This is an interactive website to engage citizens in the monitoring of tick bites in Belgium, launched in 2015 [28]. People reporting a tick bite on the website were also automatically invited to send in the tick they removed. The online questionnaire included questions on the probable geographical area where the bite occurred (postal code), the type of environment (wood, garden, natural park, etc.), the type of activity that was carried out (professional, leisure such as gardening, walking or playing, and other), as well as the age of the bitten person. Filling in the questionnaire generated a unique identifier that had to be copied on the paper containing the tick. Sending the ticks to Sciensano by postal mail was free of charge for the participants. At their arrival, ticks were stored at − 20 °C until the end of the collection period. Ticks that were not removed from humans (based on information provided in the questionnaire) or with missing information on the geographical location of the bite were discarded.

Ticks were identified morphologically to the species level and developmental stage using standard taxonomic keys [29]. Specimens that could not be identified due to extensive damage induced by the removal from the skin or from the tape were not included in the study. When multiple ticks were received from the same person at the same time, only one randomly selected tick (adult or nymph) was included to avoid oversampling of some geographical areas with a high density of ticks.

### Pathogen detection

Individual nymph and adult ticks were homogenised in Minimum Essential Media (Life Technologies, Merelbeke, Belgium) using a TissueLyser (3 min, 25 Hz) and a 5 mm metal bead. Larvae were pooled by month of the tick bite. Nucleic acids were extracted from the homogenate, using the MagMAX Total Nucleic Acid Isolation Kit and the MagMAX Express-24 Purification System (Life Technologies, Merelbeke, Belgium), according to the manufacturer’s instructions. The extracted DNA was stored at − 20 °C until a multiplex real-time PCR assay was performed on the individual nymphs and adult ticks and the pooled larvae, for molecular detection and species identification of *Anaplasma phagocytophilum*, *Babesia* spp., *Borrelia burgdorferi* (*s.l.*), *Borrelia miyamotoi*, *Neoehrlichia mikurensis* and *Rickettsia helvetica*. *Dermacentor* ticks were also screened for *R. raoultii*. The qPCR- and PCR-based approaches used were as exactly described in previous studies [26, 30, 31]. DNA from the samples that were positive from the *B. burgdorferi* (*s.l.*) qPCR, were amplified by conventional PCR, targeting the 5S-23S ribosomal RNA intergenic spacer region (IGS) of *B. burgdorferi* (*s.l.*). If the PCR was successful showing a clear band on the gel, the DNA was cleaned with ExoSAP-IT® PCR Product Cleanup Reagent (Applied Biosystems, Foster City, CA, USA) and sent to sequencing by BaseClear (Leiden, The Netherlands). The chromatographs of the sequences were visually inspected and the primers sites were trimmed in Bionumerics software version 7.6 (Applied Math, Sint-Martens-Latem, Belgium). Our sequences were used to identify the *B. burgdorferi* (*s.l.*) genospecies by comparison to sequences of known genospecies from GenBank as described previously [32]. To minimize cross-contamination and false-positive results, negative controls were included in each batch tested by PCR. In addition, DNA/RNA extraction, PCR mix preparation, sample addition and PCR analyses were performed in separated air-locked dedicated labs. Positive controls were based on plasmids containing the primer-probe-primer sequences for the target qPCR plus unique nucleotide codes between the primer and the probes. These constructs enable us to distinguish potential contaminations of samples with positive controls. Negative processing controls (50 µl distilled water) are taken along during the whole DNA extraction and qPCR procedure and negative qPCR controls (distilled water) are also taken along with the positive controls.

In addition, TBEV qRT-PCR was performed on RNA extracts, following the method described by Briggs et al. [33]. Briefly, 5 μl of RNA was mixed with the PCR master mix Kit qPCR Sqcript XLT one-step RT-QPCR (Quanta, Houston, TX, USA), 0.4 μM of the primers RH TBE Rev, primer RH TBE Fwd and primers for r18S detection (VETINHF2 and VETINHR1), 0.2 µM of TBE probe, 0.08 µM of the r18S probe and nuclease-free water to obtain a final volume of 25 μl. The amplification was performed on MxPro3005 (Stratagene system, San Diego, CA, USA) according to the following program: 30 min at 50 °C (reverse transcription) followed by 2 min at 95 °C and 45 cycles of 15 s at 95 °C (denaturation), 30 s at 55 °C (annealing and extension) and a final step of 30 s at 72 °C.

### Statistical analyses

Statistical analyses were performed in STATA 13 (Statcorp College Station, TX, USA). Co-infections and differences in pathogen prevalence by tick stage, localisation, season, type of environment, type of activity and age classes of the persons bitten were statistically analysed by Pearsonʼs Chi-square tests and Fisher’s exact tests, when appropriate. *P-*values < 0.05 were considered statistically significant.

## Results

**Fig. 1:**
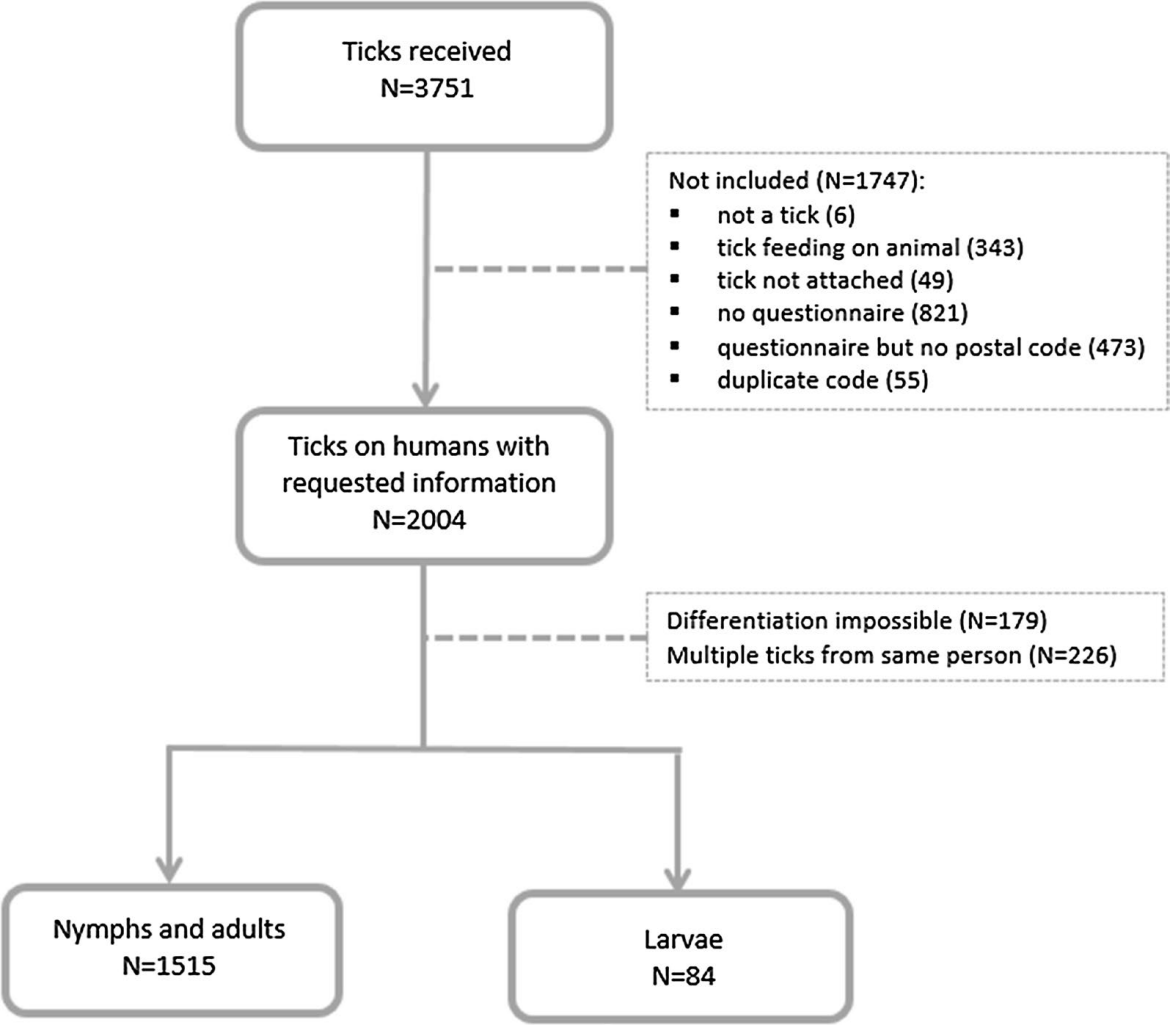
Overview of the amount of ticks at the different steps of inclusion

From April 1st till October 31st 2017, a total of 3751 ticks were sent to Sciensano, of which 2004 were (presumably) collected from humans and had information on the place of the bite. After removal of unidentifiable ticks and multiple ticks from the same person, the final sample consisted of 1225 nymphs (80.9%) and 290 adult ticks, of which 248 were females (16.4%) and 42 males (2.7%) (Table 1). In addition, 84 larvae, pooled over 7 months, were included. An overview of the inclusion flow is shown in Fig. 1.

**Table 1:** Pathogen prevalence in feeding ticks on humans by developmental stage

Tick stage	No. of ticks (%)	*B. burgdorferi* (*s.l.*)	*A. phagocytophilum*	*Babesia* spp.	*B. miyamotoi*	*N. mikurensis*	*R. helvetica*	TBEV
Nymph								
#	1225 (80.9)	151	20	17	32	37	84	0
% (95% CI)		12.3 (10.6–14.3)	1.6 (1.1–2.5)	1.4 (0.9–2.2)	2.6 (1.9–3.7)	3.0 (2.2–4.1)	6.8 (5.6–8.4)	0 (0–0.2)
Female								
#	248 (16.4)	51	8	4	3	4	18	0
% (95% CI)		20.6 (15.0–26.1)	3.2 (1.6–6.3)	1.6 (0.6–4.2)	1.2 (0.4–3.7)	1.6 (0.6–4.2)	7.3 (4.6–11.2)	0 (0–1.2)
Male								
#	42 (2.7)	8	0	1	1	2	1	0
% (95% CI)		19.1 (10.6–33.9)	0 (0–6.9)	2.4 (0.3–15.4)	2.4 (0.3–15.4)	4.8 (1.2–17.4)	2.4 (0.3–15.4)	0 (0–6.9)
Total								
#	1515 (100)	2101	28	22	36	43	103	0
% (95% CI)		3.9 (12.2–15.7)	1.8 (1.3–2.7)	1.5 (1.0–2.2)	2.4 (1.7–3.3)	2.8 (2.1–3.8)	6.8 (5.8–8.3)	0 (0–0.2)

Almost all nymphs and adult ticks belonged to *I. ricinus* (99.0%). Other tick species identified were *I. hexagonus* (0.7%, four nymphs and six females) and *D. reticulatus* (0.3%, three females and two males).

Ticks were collected from persons aged less than one year to 77 years, with a median age of 45 years. The number of ticks was well distributed over all age categories, except for the age group 15 to 24 years-old, representing only 5.5% of the specimens (Table 2). For 80.5% of the included ticks, the bite occurred in one of the four eastern provinces (Antwerp, Limburg, Liège, Luxemburg) or in the central provinces (Brabant) of Belgium.

**Table 2:** Pathogen prevalence in feeding ticks on humans according to demographic characteristics

Characteristic	*B. burgdorferi* (*s.l.*) % pos (95% CI)	*A. phagocytophilum* % pos (95% CI)	*Babesia* spp. % pos (95% CI)	*B. miyamotoi* % pos (95% CI)	*N. mikurensis* % pos (95% CI)	*R. helvetica* % pos (95% CI)
Age of the person bitten (in years)						
< 15 (*n* = 306)	15.4 (11.7–19.9)	1.6 (0.7–3.9)	0.7 (0.2–2.6)	2.9 (1.5–5.6)	3.9 (2.2–6.8)	7.2 (4.8–10.7)
15–24 (*n* = 84)	9.5 (4.8–18.0)	1.2 (0.2–8.1)	3.6 (1.1–10.6)	1.2 (0.2–8.1)	2.4 (0.6–9.1)	8.3 (4.0–16.5)
25–44 (*n* = 353)	14.2 (10.9–18.2)	2.6 (1.3–4.8)	1.1 (0.4–3.0)	2.5 (1.3–4.8)	3.1 (1.7–5.5)	5.9 (3.9–9.0)
45–64 (*n* = 506)	12.5 (9.5–15.6)	2.2 (1.2–3.9)	2.2 (1.2–3.9)	2.0 (1.1–3.6)	3.0 (1.8–4.9)	6.9 (5.0–9.5)
≥ 65 (*n* = 266)	15.8 (11.9–20.7)	0.8 (0.2–2.3)	0.8 (0.2–3.0)	2.6 (1.3–5.4)	1.1 (0.4–3.4)	6.8 (4.3–10.5)
Region						
Brussels (*n* = 20)	0	0	0	5.0 (0.7–29.3)	0	5.0 (0.7–29.3)
Flanders (*n* = 881)	14.2 (12.0–16.7)	2.2 (1.4–3.3)	1.4 (0.8–2.4)	2.5 (1.6–3.8)	3.0 (2.0–4.3)	7.6 (6.0–9.6)
Wallonia (*n* = 614)	13.8 (11.3–16.8)	1.5 (0.8–2.8)	1.6 (0.9–3.0)	2.1 (1.2–3.6)	2.8 (1.7–4.4)	5.7 (4.1–7.8)
Season						
April-June (*n* = 929)	14.5 (12.4–17.0)	2.3 (1.5–3.4)	1.4 (0.8–2.4)*	2.4 (1.6–3.6)	3.1 (2.2–4.5)	6.2 (4.9–8.0)
July-August (*n* = 484)	12.4 (9.7–15.6)	1.4 (0.7–3.0)	0.8 (0.3–2.2)**	2.9 (1.7–4.8)	2.5 (1.4–4.3)	7.6 (5.6–10.4)
September-October (*n* = 102)	14.7 (9.0–23.0)	0	4.9 (2.0–11.3)	0	2.0 (0.5–7.6)	7.8 (4.0–15.0)
Type of environment						
Wood/Forest (*n* = 513)	14.2 (11.5–17.5)	2.1 (1.2–3.8)	1.4 (0.7–2.8)	2.1 (1.2–3.8)	3.7 (2.4–5.7)	6.4 (4.6–8.9)
Garden (*n* = 666)	13.4 (11.0–16.2)	1.2 (0.6–2.4)	1.4 (0.7–2.6)	2.9 (1.8–4.4)	2.0 (1.1–3.3)	7.2 (5.5–9.4)
Nature reserve, not forest (*n* = 102)	18.6 (12.2–27.4)	4.9 (2.0–11.3)	2.0 (0.5–7.6)	2.0 (0.5–7.6)	3.9 (1.5–10.0)	5.9 (2.7–12.5)
Grassland, agricultural field (*n* = 56)	10.7 (4.9–22.0)	1.8 (0.2–11.8)	5.4 (1.7–15.5)	0	1.8 (0.2–11.8)	7.1 (2.7–17.7)
Other (*n* = 38)	15.8 (7.2–32.2)	0	0	2.6 (0.4–16.8)	0	15.8 (7.2–31.2)
Activity of person bitten						
Leisure (*n* = 1336)	14.2 (12.4–16.2)	1.7 (1.1–2.6)	1.4 (0.9–2.2)	2.4 (1.8–3.5)	2.6 (1.9–3.6)	7.1 (5.8–8.6)
Professional (*n* = 50)	16.0 (8.1–29.0)	6.0 (1.9–17.2)	2.0 (0.3–13.1)	4.0 (1.0–14.8)	6.0 (1.9–17.2)	2.0 (0.3–13.1)
Other (*n* = 47)	10.6 (4.5–23.3)	2.1 (0.3–13.9)	0	0	4.3 (1.0–15.7)	6.4 (2.0–18.2)

*Note*: Bold indicates statistically significant difference

* Statistically different (*P* = 0.01) compared to September-October

** Statistically different (*P* = 0.002) compared to September-October

*Abbreviation*: % pos: % positive

**Fig. 2:**
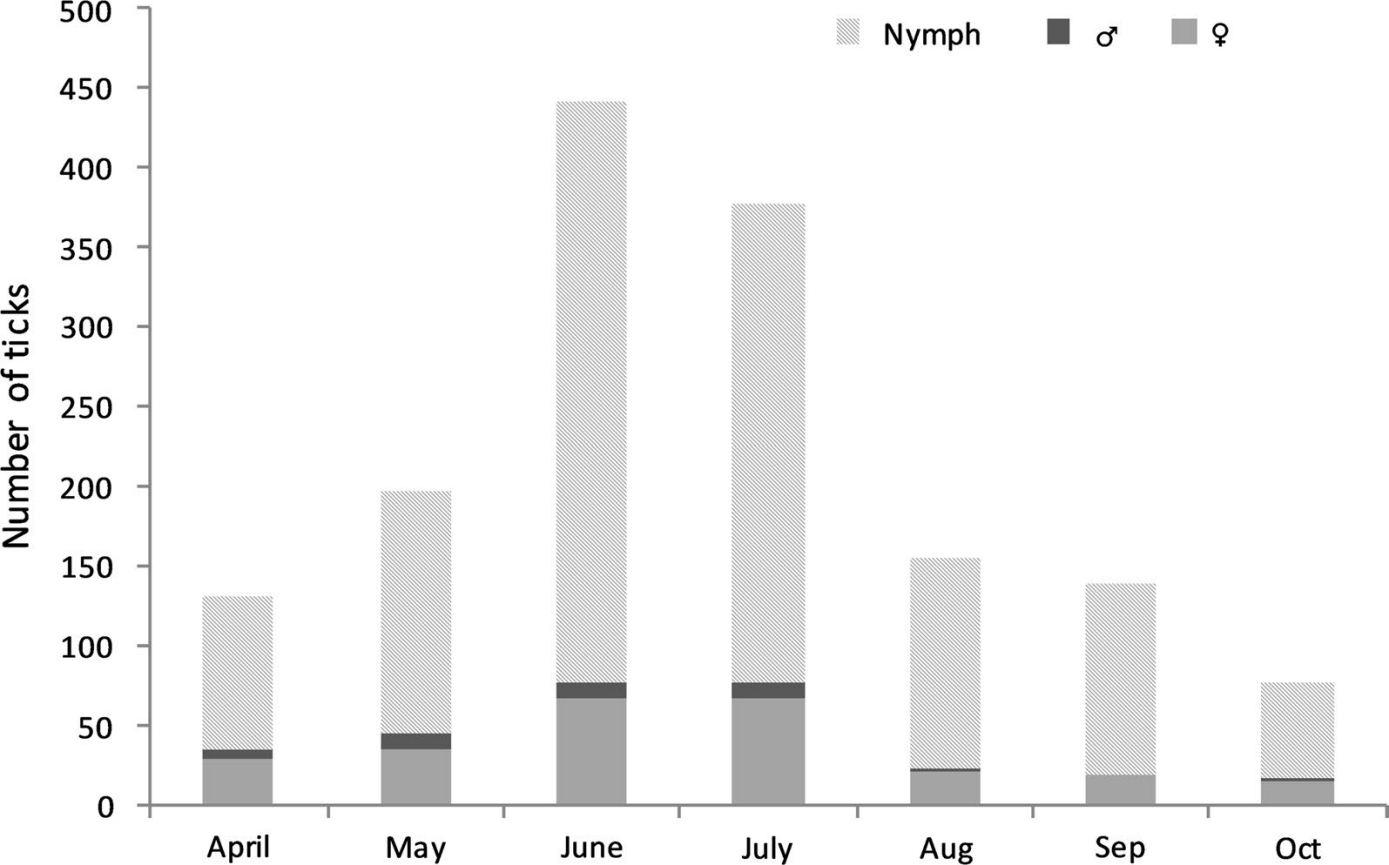
Number of ticks included in the study, by stage and month

*Ixodes ricinus* ticks were mostly sent in the months of June and July (Fig. 2). The proportion of nymphs over the total number of ticks increased from 74.8% in April to 87.5% in August (*χ*^2^ = 7.61, *df* = 1, *P* = 0.006), followed by a decrease to 75.0% in October (*χ*^2^ = 4.27, *df* = 1, *P* = 0.04). There was no significant difference in proportion of nymphs by age group (*χ*^2^ = 4.65, *df* = 4, *P* = 0.326). The median number of larvae collected by month was 10, with a minimum of 1 larva (in April) and a maximum of 23 (in July). *Ixodes hexagonus* tick bites were mostly reported between June and September (80%), whereas *D. reticulatus* ticks were sent in April and May only.

*Borrelia burgdorferi* (*s.l.*) was detected in 13.9% (95% CI: 12.2–15.7%) of nymphs and adult *Ixodes* ticks. Nymphs (12.3%) were less often infected than adults (20.3%, *χ*^2^ = 12.63, *df* = 1, *P* < 0.001) (Table 1). No *B. burgdorferi* (*s.l.*) was found in the pools of larvae. The *Borrelia* genospecies could successfully be identified for 70% of the qPCR-positive *B. burgdorferi* (*s.l.*) samples. The most frequently detected genospecies was *B. afzelii* (52.4%), followed by *B. garinii* (21.1%), *B. valaisiana* (14.3%), *B. spielmanii* (6.8%), *B. burgdorferi* (*s.s.*) (4.8%) and *B. bavariensis* (0.6%).

None of the ticks were infected with TBEV. The prevalence of other pathogens in *Ixodes* ticks ranged between 1.5–2.8%, except for *R. helvetica*, with an infection rate of 6.8% (95% CI: 5.8–8.3%) (Table 1). For the *Babesia* samples (*n* = 22), four species were identified: *B. venatorum* (77.3%), *B. divergens* (9.1%), *B. microti* (9.1%) and *B. capreoli* (4.5%). Out of the five *D. reticulatus* ticks, two were infected by *R. raoultii* (see Additional file 1: Table S1).

There were no significant differences in infection rates between developmental stages. Out of the seven pools of larvae, five were infected, one with *B. miyamotoi* (in May) and the four others with *R. helvetica* (April, June, July and August).

A detailed overview of infection rates by different characteristics is shown in Table 2. No significant differences were found in pathogen prevalence by age class of the person bitten, by region (Flanders, Wallonia or Brussels), season, type of environment (wood, garden, natural park, field or other such as dunes or a golf court) and type of activity (professional, leisure or others, such as short stays in the garden for hanging up laundry or at school). The only exception was a significantly higher prevalence of *Babesia* spp. in ticks in autumn (September and October) compared to other seasons (*χ*^2^ = 9.82, *df* = 2, *P* = 0.007).

**Fig. 3:**
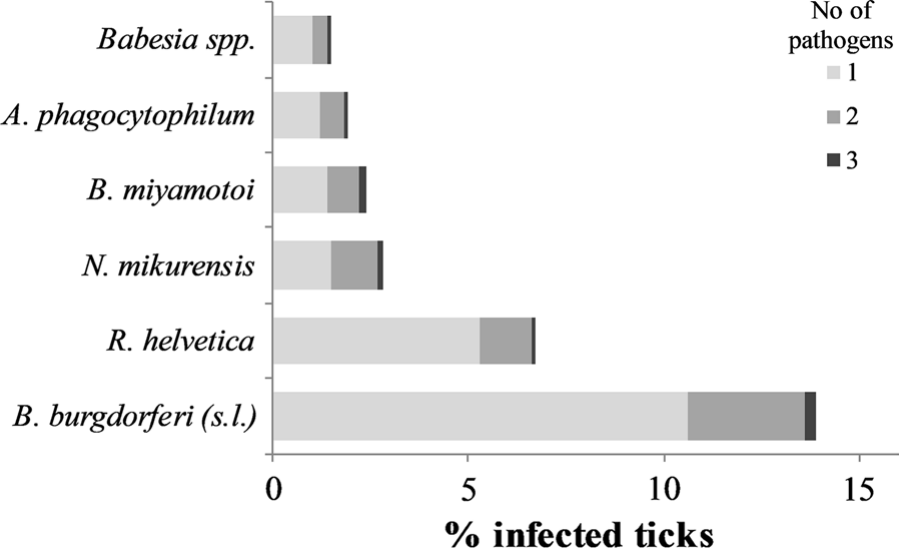
For each pathogen, proportion of feeding ticks on humans infected with the pathogen (referred to as 1) and co-infected with one or two more pathogens (referred to as 2 and 3, respectively) are shown (*n* = 1515)

Co-infections were found in 3.9% (59/1515) of the examined ticks (Fig. 3) and only in *I. ricinus* ticks; 3.6% (55/1515) of nymphs and adult ticks carried two pathogens and four ticks (0.3%) were infected with three pathogens. The most common co-infection in ticks was *B. burgdorferi* (*s.l*.) + *N. mikurensis* (17 infected ticks out of 381), followed by *B. burgdorferi* (*s.l*.) + *R. helvetica* (14 infected ticks out of 381); ticks infected with either *B. miyamotoi* or *N. mikurensis* showed the highest proportion of co-infections with other pathogens (Table 3 and Fig. 3). Infections with *N. mikurensis* or *B. miyamotoi* are more likely to occur as co-infection with *B. burgdorferi* (*s.l.*).

**Table 3:** Number of ticks presenting a co-infection for different pathogen combinations

	*B. burgdorferi* (*s.l.*)	*A. phagocytophilum*	*Babesia* spp.	*B. miyamotoi*	*N. mikurensis*
*B. burgdorferi* (*s.l.*) (n = 210)	–				
*A. phagocytophilum* (n = 28)	7	–			
*Babesia* spp. (*n* = 22)	2	0	–		
*B. miyamotoi* (*n* = 36)	13*	1	1	–	
*N. mikurensis* (*n* = 43)	17**	0	2	0	–
*R. helvetica* (*n* = 105)	14	3	3	3	1

*P = 0.001, **P < 0.001

## Discussion

To our knowledge, no study of the presence of pathogens has been carried out on feeding ticks collected from human hosts in Belgium, but several such studies have been carried out, on one or some pathogens, in other European countries, such as in the Netherlands, Finland, France, Poland, Italy, Germany and Sweden [30, 34–40]. In most studies, ticks were removed by physicians, whereas in our study they were removed by citizens and sent by postal mail. The call to citizens to participate to the collection of ticks, through a single press release, generated a marked interest and resulted in a sample of over 3700 ticks in only half a year. If the information was not specified otherwise in the questionnaire or on the paper containing the tick, we assumed that the specimen was removed from a human. The number of ticks removed from other hosts is probably limited and is unlikely to impact our results. People participating to the study seem to have good knowledge on ticks, since only six specimens received were no ticks but other arachnids or vegetal parts.

Although more than half of the ticks received could not be included in the study due to missing information or damage, our final sample covered ticks of all developmental stages, collected all over the country over several months. Citizen science participation was also used to collect ticks (from humans and animals) *via* mail and zip-locked plastic bags in a study in the USA in 2016–2017, with submission of over 16,000 ticks [41]. Both studies demonstrate that citizen science can be an effective tool to collect ticks for surveillance or research, at a relatively low cost.

The ticks removed from humans in Belgium were almost exclusively *I. ricinus* (99%), the most widespread and abundant tick species transmitting pathogens causing tick-borne disease in human in Europe. Besides *I. ricinus*, a few specimens of *I. hexagonus* and *D. reticulatus* were collected. A review by Obsomer et al. [42] on the spatial distribution of tick species in Belgium, based on new tick collections and a literature and “grey datasets” search between 1989 and 2012, showed that these three species were reported most frequently. *Ixodes ricinus* and *I. hexagonus* were found to be present in all the provinces, while *D. reticulatus* showed a more patchy distribution. In our sample, two out of the five specimens of the latter were sent from De Panne, on the border with France, where the tick has repeatedly been reported previously [42, 43]. *Ixodes hexagonus* was also removed from patients in Germany and *D. reticulatus* in Poland [37, 39].

The number of ticks per province included in the study is consistent with the spatial distribution of human tick bite occurrence and Lyme borreliosis in the country, with a higher number of reports in the eastern part [18, 28].

The observed predominance of nymphs (81%) was higher than reported in several other European studies on ticks removed from humans, where proportions of nymphs ranged between 41% (Italy) and 70% (Sweden) [38, 40]. In a citizen-based tick reporting study in Great Britain, the proportion of nymphs removed from humans was also 81% [44].

For both, *I. ricinus* and *D. reticulatus,* a bimodal questing activity pattern has been described, with peaks in spring (March-May) and in late summer or autumn (mid-August-November) [45–47]. *Ixodes hexagonus* appears to show less marked seasonal changes than *I. ricinus* [44]. In our study, the peak in number of ticks sent in June and July corresponds to a higher exposure during these months (holiday period, warmer weather) rather than a peak of questing activity.

The observed prevalence of *B. burgdorferi* (*s.l.*) infection in *Ixodes* ticks in our study (13.9%) is in line with the overall mean prevalence of 13.7% (range: 0–49.1%) in Europe, reported by a meta-analysis of surveillance data in 2011 [48]. Previous studies in Belgium on questing *I. ricinus* ticks reported high variability in infection rates ranging from 2.8% to 37% [22, 24, 25]. However, these studies were often limited to ticks collected over a short time period, at one spot or in a small geographical area, whereas the distribution and prevalence of pathogens in ticks is known to show important variations, both temporally and spatially [49]. The observed significant lower infection rates in nymphs compared to adults (12.3% and 20.3%, respectively) is in accordance with previous reports and can be explained by the fact that host-seeking adult ticks have had two blood meals from different hosts and therefore have a higher probability of acquiring bacteria from infected hosts [48–50]. Although in our study larvae were not infected by *B. burgdorferi* (*s.l.*), detection of the spirochetes in larvae has previously been described, at a low prevalence [50]. In line with other studies in Belgium and neighbouring countries, the rodent-associated *B. afzelii* was the most common *Borrelia* genospecies (52.4%), followed by *B. garinii* (21.1%) [24, 39, 51–53]. Both are dominant genospecies in ticks in Europe [54]. *Borrelia valaisiana*, *B. spielmanii* and *B. burgdorferi* (*s.s.*) represented 14.3%, 6.8% and 4.8% of genospecies, respectively. *Borrelia bavariensis* (0.7%) was previously described in Belgium in one study only, in 17 out of 1203 ticks (1.4%) removed from hedgehogs [26].

Except for TBEV, the other tick-borne pathogens studied were all detected in the tick sample, although at a lower prevalence, ranging between 1.5% (*Babesia* spp.) and 6.8% (*R. helvetica*). *Anaplasma phagocytophilum* was detected in 1.8% of nymphs and adult ticks. This is in line with the relatively low number of human cases of anaplasmosis diagnosed in Belgium, compared to Lyme borreliosis. However, many cases of the disease probably remain undiagnosed [11]. Studies on questing ticks reported a similar prevalence (1.2–3.0%) [21, 55].

All three *Babesia* species causing disease in humans in Europe, *B. divergens*, *B. venatorum* (sp. EU1) and *B. microti*, have been detected in our study, although at low prevalence. In ticks feeding on animals (cats and dogs, 1.3%; wild cervids, 2.7%), equally low rates have been reported [23, 56]. A study on bovine *Babesia* spp. with targeted sampling in areas with known babesiosis reported higher values: 7.9% in questing ticks and 14.6% in feeding ticks on cattle [55].

The prevalence of *B. miyamotoi* in ticks removed from humans (2.4%) is slightly higher than results from earlier studies on questing ticks (1.1–1.6%, 2010–2014) [20, 21]. The absence of human cases in Belgium up to now might be due to the absence of routine laboratory testing and low clinical awareness.

The absence of diagnoses of neoehrlichiosis in Belgium might also be related to low awareness and lack of diagnostic testing, since 2.8% of nymphs and adult ticks examined here were infected with *N. mikurensis*. A recent review of studies on the presence of *N. mikurensis* in *I. ricinus* ticks in Europe reported prevalence in ticks removed from humans between 0.5% in Italy and 8.1% in Germany [14]. In questing ticks, much higher infection rates have been reported, up to 17% in Norway; in Belgium, only one study has been published, reporting a very low prevalence of 0.4% [21]. Studies in the Netherlands and Norway using molecular detection techniques found *N. mikurensis* in 1.4% and 10.0%, respectively, of patients presenting with an erythema migrans after a tick bite [30, 57]. However, there is no evidence of a causal relation. The pathogenicity of *N. mikurensis* should be further investigated.

*Rickettsia helvetica* has been detected in *I. ricinus* ticks in at least 24 European countries, but human infection with this species has been described (as a relatively mild, self-limited illness) in a few countries only [17]. Since 2005, no definitive, convincing cases have been published [17]. It is therefore difficult to relate the relatively high prevalence of *R. helvetica* (6.8%) in Belgian ticks to a risk for human disease. On the other hand, the pathogenic species *R. raoultii*, reported in many European countries, was identified for the first time in our study, in two out of the five *D. reticulatus* ticks collected. However, this tick species only sporadically bites humans. In studies on questing ticks and ticks that fed on animals (dogs and cats in Belgium and songbirds in Belgium/The Netherlands), *R. helvetica* was found more often than in our study (16.9%, 14.1% and 22% of *Ixodes* ticks, respectively) [19, 21, 58].

The prevalence of TBEV in ticks was examined for the first time in Belgium in this study. The absence of the virus in our sample does not mean that the virus is not present, as it has been shown through studies in animals and possible recent human autochthonous infections in Belgium [9, 10]. An extensive study in Poland and Germany showed that the virus prevalence in ticks does not correlate with increased risk for humans [59]. Additional surveillance methods, such as seroprevalence studies in animals, should be further implemented.

Co-infections in ticks are frequently reported. They may result from co-feeding of infected ticks on one host, from a blood meal on one host carrying several pathogens or from blood meals on different hosts. Most of the co-infections reported are associations between different *Borrelia* genospecies, which could not be differentiated in our study because the approach used was unable to univocally identify infections with more than one *B. burgdorferi* (*s.l.*) genospecies. In a study on ticks feeding on humans in Italy, 5.7% of *Ixodes* ticks were infected with more than one pathogen and a study on questing *I. ricinus* ticks in Romania reports co-infection of one *Borreli*a spp. with another pathogen in 3.7% of ticks, similar to the 3.9% we observed [38, 60]. As in our study, the most frequent dual co-infections in Romania were between *Borrelia* spp. and *Rickettsia* spp. and between *Borrelia* spp. and *N. mikurensis* [60]. In ticks on songbirds in Belgium and the Netherlands, the occurrence of *B. burgdorferi* (*s.l.*) was also positively correlated with the occurrence of *N. mikurensis*, suggesting transmission facilitation due to interactions between pathogens [58]. Co-infection of *Borrelia* spp. with *Babesia* spp., suspected to enhance the severity of Lyme borreliosis, was rare in our study (2/1515 ticks) and not statistically significant [61, 62]. Further research is needed to investigate the possible effect of co-infections on disease in humans.

No statistically significant associations were observed for pathogen prevalence according to the age of the person bitten, the region or province in Belgium, the season (except for a higher prevalence of *Babesia* species in autumn), the type of environment or the type of activity during which the bite occurred. However, for all pathogens, the prevalence seems to be higher in ticks collected during a professional activity than a leisure exposure and some pathogens (*B. burgdorferi* (*s.l.*), *A. phagocytophilum* and *N. mikurensis*) tend to be more common when the tick bite occurred in nature reserves or woods and forests, compared to gardens and fields. This could be related to the higher density of animal reservoirs in these areas. A larger sample size might have contributed to detecting a statistical significant relation.

## Conclusions

A citizen-based collection method, based on an existing notification tool for tick bites, allowed to collect an important sample of ticks across the country, covering the whole tick season and at low cost. The present study serves as a status survey for the infection rate of several tick-borne pathogens in Belgian ticks that were attached to humans and allows informing the health authorities on emerging tick-borne disease risks. In comparison to studies on questing ticks, this approach is more likely to reflect the actual tick-borne disease risk (pathogen exposure) for humans. Except for TBEV, all the pathogens tested were detected in the tick study sample, yet, to a different extent. This confirmation can help to raise awareness among citizens and health professionals in Belgium on possible diseases other than Lyme borreliosis in persons presenting fever or other non-characteristic symptoms after a tick bite. A new study is planned, to allow follow-up and assessment of potential variation in infection prevalence and thus infection risk for humans, over time and space.

## Supplementary information

**Additional file 1: Table S1.** Dataset of qPCR results and DNA sequences.

## Data Availability

Data supporting the conclusions of this article are included within the article and its additional file (the dataset of qPCR results and the DNA sequences). Representative sequences of this study are submitted to the GenBank database under the accession numbers MN114277-MN114278. The dataset on the tick collection is available from the corresponding author upon reasonable request.

## Abbreviations

*s.l.*: *sensu lato*
*s.s.*: *sensu stricto*
TBEV: tick-borne encephalitis virus
